# Imaging of Gastrointestinal Tract Ailments

**DOI:** 10.3390/jimaging9060115

**Published:** 2023-06-08

**Authors:** Boyang Sun, Jingang Liu, Silu Li, Jonathan F. Lovell, Yumiao Zhang

**Affiliations:** 1 Key Laboratory of Systems Bioengineering, School of Chemical Engineering and Technology, Frontiers Science Center for Synthetic Biology (Ministry of Education), Tianjin University, Tianjin 300350, China; boyangsun@tju.edu.cn (B.S.); jingang_liu@tju.edu.cn (J.L.); lisilu_0122@tju.edu.cn (S.L.); 2 Department of Biomedical Engineering, The State University of New York at Buffalo, Buffalo, NY 14260, USA

**Keywords:** gastrointestinal imaging, multimodal imaging, inflammatory bowel disease, appendicitis, intestinal obstruction, Meckel’s diverticulum

## Abstract

Gastrointestinal (GI) disorders comprise a diverse range of conditions that can significantly reduce the quality of life and can even be life-threatening in serious cases. The development of accurate and rapid detection approaches is of essential importance for early diagnosis and timely management of GI diseases. This review mainly focuses on the imaging of several representative gastrointestinal ailments, such as inflammatory bowel disease, tumors, appendicitis, Meckel’s diverticulum, and others. Various imaging modalities commonly used for the gastrointestinal tract, including magnetic resonance imaging (MRI), positron emission tomography (PET) and single photon emission computed tomography (SPECT), and photoacoustic tomography (PAT) and multimodal imaging with mode overlap are summarized. These achievements in single and multimodal imaging provide useful guidance for improved diagnosis, staging, and treatment of the corresponding gastrointestinal diseases. The review evaluates the strengths and weaknesses of different imaging techniques and summarizes the development of imaging techniques used for diagnosing gastrointestinal ailments.

## 1. Introduction

Gastrointestinal [GI] tract ailments encompass a common and diverse group of diseases that are prevalent in various populations, with inflammatory bowel disease being a common chronic and recurrent condition affecting over 1.2 million Americans and showing an increasing incidence in other populations [1,2]. In the past, endoscopy and fluoroscopy were the primary diagnostic tests for GI tract assessment. However, these techniques have limitations in evaluating the submucosal layers of the bowel wall and are invasive, which may be suboptimal when repeated studies are needed over time [3,4,5,6]. Currently, cross-sectional imaging techniques such as computed tomography (CT) and magnetic resonance imaging (MRI) are considered the preferred modalities for assessing the bowel wall. Ultrasound has also demonstrated a limited role in assessing the bowel wall. Numerous studies have demonstrated the performance of imaging GI tract modalities such as computed tomography (CT) [7,8], magnetic resonance imaging (MRI) [3,9], and photoacoustic tomography (PAT) [10,11,12,13,14]. However, these techniques provide anatomic imaging but lack functional information. Molecular imaging, which is a diverse field encompassing various modalities, has the potential to provide additional functional information that can aid in guiding management decisions. Recent developments in molecular imaging have focused on improving and combining modalities to create more sophisticated imaging approaches. Imaging of the GI tract plays a crucial role in the diagnosis and treatment of chronic GI diseases. Multiple imaging modalities are available for GI tract imaging, including MRI, PET, SPECT, ultrasound (US), and PAT. On one hand, these imaging modalities can provide us with accurate information; on the other hand, these come with inherent limitations such as limited spatial resolution, poor sensitivity, contrast agents, or radiation exposure. This provides the impetus for a review of commonly used modalities in the preclinical and clinical setting to determine the best-suited modality for each of the GI tract diseases.

### 1.1. X-ray/CT

X-ray and computed tomography (CT) are imaging techniques that have seen significant advancements in recent years. These advancements have led to improved sensitivity, reduced exposure time, and decreased patient radiation dose in X-ray imaging. CT, in particular, has become a widely used imaging modality for diagnosing diseases of the gastrointestinal (GI) tract due to its high diagnostic accuracy for most GI indications. CT, which involves the emission and detection of X-rays, offers high per-slice acquisition rates and scans with a total exposure of less than 1 mSv, alleviating concerns about radiation exposure compared to previous techniques [15]. CT has become commonly used for detecting structural abnormalities such as tumors and fibrosis, as well as diagnosing conditions of the chest, abdomen, and upper GI tract [2,16]. In contrast to X-ray imaging, which has seen a decline in use for GI tract indications, CT has become a preferred choice for many clinicians due to its superior diagnostic accuracy and reduced radiation exposure. The advancements in CT technology have made it a valuable tool in the diagnosis of GI diseases, allowing for improved visualization and assessment of the GI tract with minimal radiation risk to patients.

### 1.2. MRI

Magnetic resonance imaging (MRI) is another powerful imaging modality that has gained popularity in recent years for evaluating diseases of the GI tract [17,18]. Unlike X-ray and computed tomography (CT) which utilize ionizing radiation, MRI employs powerful magnets and radio waves to generate detailed images of the internal structures of the body without exposing patients to harmful radiation [19,20]. MRI offers several advantages for GI imaging, including its ability to provide high-resolution images of soft tissues, such as the GI tract, allowing for detailed visualization of anatomical structures and functional assessment [21]. MRI can also provide valuable information about blood flow, inflammation, and tissue characteristics, making it particularly useful for evaluating conditions such as Crohn’s disease, ulcerative colitis, and tumors in the GI tract [22]. MRI is considered especially valuable in evaluating the small bowel, as it can provide detailed images of the intestinal wall, detect inflammation, and assess the presence of strictures or fistulas [23]. Additionally, MRI with contrast enhancement using gadolinium-based contrast agents can help improve the visualization of lesions and enhance the diagnostic accuracy of GI diseases [22]. Although MRI has some limitations, such as its relatively higher cost and longer acquisition times compared to other imaging modalities, its non-invasive nature, lack of ionizing radiation, and excellent soft tissue contrast make it a preferred choice for many clinicians in the evaluation of GI diseases, especially in cases where detailed anatomical and functional information is required.

### 1.3. PET/SPECT

PET (positron emission tomography) and SPECT (single-photon emission computed tomography) are nuclear medicine imaging techniques that are used in combination with other imaging modalities that rely on the use of radiolabeled antibodies or markers, such as identifying body glucose uptake [24]. PET imaging involves the use of a radioactive tracer that is injected into the patient’s body while SPECT uses a gamma camera to detect the gamma rays emitted by a radioactive tracer that is injected into the patient’s body. PET is particularly useful in detecting metabolic changes in tissues, which can help identify areas of increased activity, such as tumors or areas of inflammation. SPECT is commonly used in combination with CT or MRI to provide functional and anatomical information in the evaluation of GI diseases [20,25]. PET and SPECT can be utilized in the evaluation of a wide range of GI conditions, including cancer staging, assessment of treatment response, and detection of recurrent disease [26]. They can also provide valuable information about blood flow, metabolism, and molecular changes in tissues, allowing for early detection and monitoring of disease progression. One of the advantages of PET and SPECT is their ability to detect functional changes in tissues before structural changes are evident, making them valuable tools in the early detection of GI diseases, which can help guide appropriate treatment strategies [27]. When used carefully and in combination with other imaging modalities, they can aid in early detection, assessment of treatment response, and personalized treatment planning. However, it is worth noting that PET and SPECT also have some limitations: relatively expensive imaging and potential exposure to ionizing radiation due to the use of radioactive tracers.

### 1.4. Ultrasound

Ultrasound imaging, also known as sonography, utilizes high-frequency sound waves to create real-time images or video of soft tissues inside the body. It is a rapid, low-cost, and widely available imaging tool that has the advantage of not exposing patients to ionizing radiation, making it particularly important in pediatrics. Ultrasound is commonly used for diagnosing diseases and assessing their structure and functionality [24]. Gas-filled microbubbles can serve as contrast agents in ultrasound imaging, enhancing the visualization of disease processes such as tumors or areas of inflammation by undergoing acoustic oscillations or collapsing at the target site, resulting in the generation of strong echoes or signals [28]. However, ultrasound has inherent limitations in terms of sensitivity and depth of penetration, which may impact its diagnostic accuracy in certain cases. In such situations, magnetic resonance imaging (MRI) may be preferred over ultrasound due to its higher diagnostic accuracy, making it a more reliable imaging modality [29].

### 1.5. Photoacoustic Tomography

Photoacoustic tomography (PAT) is a cutting-edge imaging technique that combines ultrasound and laser-induced photoacoustic signals to generate detailed images of biological tissues, including the GI tract, with high contrast in optical imaging and high resolution in deep tissues with acoustic imaging [30,31,32]. PAT uses laser pulses to create photoacoustic waves that are generated when absorbed light is converted into heat, leading to localized thermoelastic expansion and subsequent ultrasound waves, then detected by ultrasonic sensors, which are then analyzed to produce images. PAT is essentially optical imaging, good acoustic spatial resolution can be achieved even at imaging depths of 5–6 cm, making it suitable for imaging deeper structures in the GI tract [11,33]. PAT has high sensitivity and can detect molecular-level changes in tissues, making it a promising tool for clinical images, such as tumor diagnosis imaging [34,35], whole-body imaging of small animals [36], and various other medical applications [37].

## 2. Imaging of Gastrointestinal Diseases

### 2.1. Inflammatory Bowel Disease (IBD)

Inflammatory bowel disease (IBD) is a multifactorial and idiopathic inflammatory disorder that affects the gastrointestinal tract, presenting in two major forms: Crohn’s disease (CD) and ulcerative colitis (UC). Ulcerative colitis is typically confined to the colon, with continuous mucosal inflammation extending proximally, leading to structural changes in the crypt and inflammatory infiltration, resulting in conditions such as toxic megacolon and fulminant colitis. On the other hand, Crohn’s disease can involve any part of the gastrointestinal tract, with lesions that are usually discontinuous and characterized by transmural inflammation, leading to fibrotic strictures, fistulas, and non-caseating granulomas [38]. The mucosal immune responses in IBD are influenced by a complex interplay of genetic, environmental, and other factors [39].

To establish a primary diagnosis and provide guidance for treatment, many diagnostic tests are used, parameters assessing pathology including bowel wall thickness, mural changes, vascularization, and perienteric inflammatory changes [40]. In addition to conventional investigations such as barium studies, colonoscopy, ileal intubation, and small bowel follow-through (SBFT) and small bowel enteroclysis, novel imaging modalities are emerging for the diagnosis, follow-up, and management of patients with suspected or confirmed IBD [41]. Here, the review will focus on the scope of the imaging fields of the GI tract.

#### 2.1.1. PET Image of IBD

**Figure 1:**
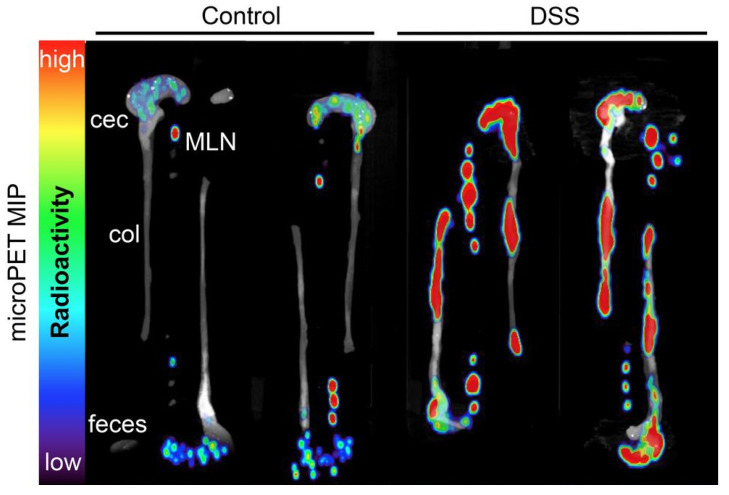
Immuno-PET images of colons, ceca, and mesenteric lymph nodes in mice. Reprinted with the permission of [50].

Unlike indirect methods including activity indexes, blood, and stool test, or invasive methods such as colonoscopy and deep enteroscopy, FDG-PET (2-(18F) fluoro-2-deoxy-D-glucose positron emission tomography) is a reliable imaging technique for diagnosing inflammatory bowel disease (IBD) was used in PET scanning to identify areas of inflammation where there is an abnormally high glycolytic rate [42]. In a study conducted by Bicik et al. in 1997 on 7 patients suspected of having IBD, FDG-PET was able to provide consistent results with clinical data, endoscopy, and histological analysis in 6 out of 7 patients (4 with Crohn’s disease and 2 with ulcerative colitis). The authors concluded that FDG-PET may be a useful non-invasive tool for identifying active IBD for long-term monitoring of patients [43]. Other studies by Holtmann et al. in 2012 [44], Berthold et al. in 2013 [45], Treglia et al. in 2017 [46] and Lovinfosse et al. in 2022 [47] also demonstrated that FDG-PET could be an effective detection tool for IBD with good sensitivity and specificity. In addition to the widely used FDG-PET, the development of 18F-FSPG PET ((4S)-4-(3-18F-fluoropropyl)-l-glutamate) has also been reported in clinical trials, demonstrating promising results for diagnosing endoscopically active inflammatory bowel disease (IBD) and remission in patients and bowel segments [48]. Specifically, 18F-FSPG PET was found to correctly identify all 9 patients with superficial or deep ulcers, indicating its potential as an accurate diagnostic tool for IBD [48]. Recently, a novel PET imaging method named ImmunoPET (PET imaging with antibody fragment probes) has attracted attention in the field of IBD imaging [49]. As shown in Figure 1, zirconium-89 (^89^Zr)-labeled anti-CD4 engineered antibody fragment [GK1.5 cDb] has been used for PET imaging of CD4^+^ T cells, a characteristic feature of IBD, successfully detecting CD4+ T cells in the colon, cecum, and mesenteric lymph nodes [50].

Similarly, immunoPET probes targeting characteristic proteins of IBD were enriched such as ^89^Zr-α-IL-1β and ^89^Zr-α-CD11b immuno-PET probe [51], as well as ^89^Zr-desferrioxamine-infliximab probe [52]. These advancements in PET imaging have laid the foundation for achieving more accurate diagnosis and treatment of IBD.

#### 2.1.2. CT and CTE of IBD

**Figure 2:**
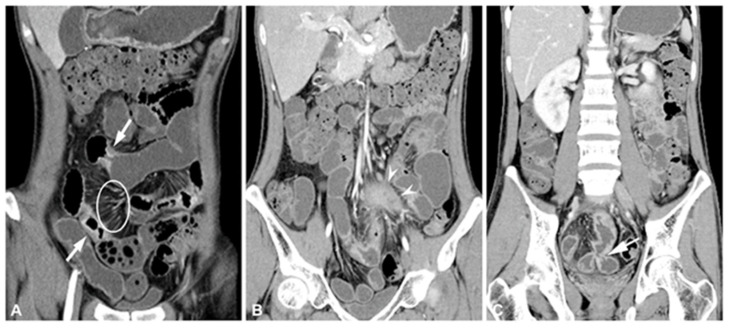
Diagnosis of inflammatory Crohn’s disease with extraenteric complications by computed tomography enterography. (**A**) Multifocal segmental stricture showing wall thickening, mural hyperenhancement, and stratification shown by arrows and engorged vasa recta, comb sign highlighted by a circle. (**B**) Mesenteric abscess is shown by arrowheads. (**C**) Adjacent to the enteroenteric fistula shown by the arrow. Reprinted with permission of [57].

Computer tomography (CT) is a well-established diagnostic tool for three-dimensional imaging of the gastrointestinal tract, organs, and tumorous tissue based on X-ray technology [53]. CT provides crucial information on pathological changes involving a mesenteric attachment, the bowel wall, and adjacent structures [54]. The CT features of inflammatory bowel disease (IBD) typically include wall thickness, sub-mucosal fat, isolated right colon involvement, and mesenteric fibrofatty proliferation. The clinical trial data revealed that the improved GIF algorithm can enhance the application value of low-dose CT images [55]. Introduced in 1997, CT enterography (CTE) has emerged as the imaging modality of choice for assessing acute IBD [56]. CTE has become the preferred imaging technique in clinical practice for the assessment of acute IBD [29]. This technique involves luminal distention with a large amount of oral contrast agent, followed by intravenously administered contrast agents to enhance the bowel wall and its pathology, and thin-section CT scans [12]. CTE provides important information for the diagnosis and management of IBD, including Crohn’s disease, intestinal tuberculosis (TB), and intestinal Behcet’s disease (BD) [57], as shown in Figure 2. CTE can also provide information on mural stratification and extraenteric findings such as comb sign, with combinations of bowel wall thickening and mural hyperenhancement being sensitive findings for activating inflammatory Crohn’s disease [58].

Clinical studies have shown that CTE has better sensitivity than MR enterography for distinguishing pericentric features, likely due to the increased conspicuity of the mesentery in CT enterography [59]. Meanwhile, MR enterography yielded significantly more motion artifacts than CT enterography did [60]. Nevertheless, both CT enterography and MR enterography have similar diagnostic accuracy for the diagnosis of Crohn’s disease and its complications [61]. The role of CT in the investigation of IBD is controversial [62]. On one hand, CT is suggested as an important modality for evaluating colonic inflammation, the diagnosis and management of IBD in patients with acute symptoms [63], and the management of patients with known IBD [64], and serves as an essential diagnostic method in patients with CD [65]. CT enterography is also preferred as a baseline study for most patients [66,67,68]. On the other hand, CT has also been reported to be less useful for detecting early CD [64,69]. Moreover, CT is also not recommended as a primary method of diagnosis of the early stages of UC, characterized by extensive ulceration and diffuse inflammation of the mucosa, which may not be detected on CT scans. There are also concerns about the significant ionizing radiation risk associated with multi-detector CT or repetitive CT imaging, especially in patients with IBD who may require frequent imaging throughout their lives, patients with IBD receive CT exams in the emergency department or inpatient setting more than a quarter of the time, and pediatric patients with IBD are particularly at risk of radiation exposure-related cancer mortality [70,71]. Therefore, pediatric providers often limit radiation exposure in patients under 18 years of age [72]. Nevertheless, due to the capability of detecting certain complications and explaining many morphologic changes of advanced UC, CT could be used as a complementary imaging method.

#### 2.1.3. PET/CT of IBD

To provide accurate spatial resolution and anatomical information of FDG activity, CT was often married to PET, referred to as PET/CT modality. This modality, with good sensitivity and specificity [73,74], was shown to be more capable and efficacious than CT or PET alone, merging the anatomical data offered by CT and the physiological data provided by PET [74,75,76]. Das et al. conducted a study using PET/CT enteroclysis in 17 patients with newly diagnosed inflammatory diseases of the intestine. The study involved performing a low-dose whole-body PET/CT scan followed by the injection of 10 mCi of FDG 60 min later. Subsequently, after infusion of 0.5% methylcellulose through a naso jejunal catheter, PET/CT enteroclysis of the abdomen was performed. This technique was found to have a superior detection rate compared to conventional methods such as colonoscopy and barium studies, with a total of 50 segments (23 segments of the small intestine and 27 segments of the large intestine) being detected. Although this study lacks an endoscopic evaluation for comparison and follow-up study, it can be concluded that this non-invasive fused technique can detect more lesions in both the small intestine and large intestine compared to the conventional methods [77]. Another study was conducted in patients with known or suspected IBD using PET/CT imaging. Abdominal and pelvic images were acquired using a PET/CT multislice scanner, with FDG being administered intravenously at a dose of 0.14 mCi/kg, followed by a low-dose, non-contrast CT scan with 120 mA. The five bowel regions that were observed from this particular CT component included the small bowel, ascending, transverse, descending, and rectosigmoid. The study included four patients with Crohn’s disease (CD), two patients with ileal pouch-anal anastomosis surgery for ulcerative colitis (UC), and one patient with suspected IBD. The CT/PET scan was shown to contribute greatly to clinical decision-making in terms of selecting the correct treatment and therapy, whereas the standard tests underestimated the CD activity and gave inaccurate results [78]. PET imaging was necessary in addition to CT imaging to make a definitive diagnosis [79]. PET/CT has also emerged as a valuable modality for monitoring therapeutic response [80,81], in a study by Spier et al., PET/CT was conducted on 5 patients with CD (*n* = 3) or UC (*n* = 2) before and after drug treatment, revealing decreased or absent radiotracer uptake in previously active segments after treatment, correlating with symptom improvement [81].

In summary, PET/CT enables a comprehensive disease assessment and close disease monitoring that may not be achievable with other methodologies. It effectively solves the frequency limitations and high discomfort of patients of invasive surgery, the risks of dangerous radiation amount with lower sensitivity by Barium research, and the high cost for low-quality imaging of MRI. Compared to MRI, PET/CT offers superior specificity and sensitivity, as well as faster diagnosis and lower costs [74].

#### 2.1.4. MRI for IBD

**Figure 3:**
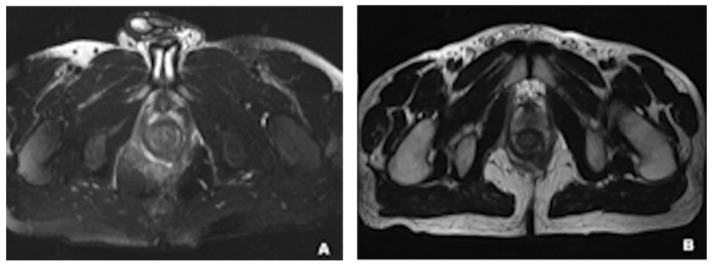
An oblique axial T2w fat-sat (**A**) and T2w (**B**) images show a complex transsphinteric fistulous tract with a “horseshoe” feature. Reprinted with permission of [87].

One concern about the use of PET/CT or CT is radiation exposure [82]. In this regard, the replacement of CT with magnetic resonance imaging (MRI) scan is a better option considering the significantly lower radiation dose of MRI, especially for chronic conditions where multiple serial examinations are needed for the assessment of the progression of the disease and therapy efficacy monitor [3,83]. Moreover, MRI offers superior soft tissue contrast resolution compared to CT, allowing for better visualization of inflammatory and fibrotic characteristics of the intestinal wall [61,84]. Pelvic MRI has become a standard imaging modality for patients with Crohn’s disease, suspected perianal involvement, as well as the condition of perianal fistulas, due to its ability to provide precise and detailed images of the sphincter complex (Figure 3) [61,85,86,87], the sensitivity and specificity of MRI are 100% and 86% for detecting fistula tracks [86]. A combination of endoanal ultrasound and MRI may further increase diagnostic accuracy [88].

Despite the time-consuming nature of MRI scans, the utilization of advanced MRI techniques can reduce the duration, and the implementation of PET/MR scanners allows for the simultaneous acquisition of PET and MR data [89,90,91]. Catalano et al. demonstrated that the PET/MRI exhibited superior diagnostic performance compared to each modality independently. Sensitivity, specificity, and diagnostic accuracy were 91.5%, 74%, and 84% for PET; 80%, 87%, and 83% for MR; and 88%, 93%, and 91% for PET/MR, respectively [91]. In addition, the advantage of MRI imaging stands out when it comes to diagnostic imaging for pediatric patients [92]. For small bowel imaging, MRI was mainly achieved by MR enterography or MR enteroclysis [23,93]. Although patients are uncomfortable with the nasojejunal intubation for MR enteroclysis compared to the oral administration of contrast agent for MR enterography, the degree of intestinal distention obtained by enteroclysis is better than the oral administration. Therefore, the preference for performing one over the other is still controversial. Some studies showed that the two techniques have similar sensitivity in the detection of active inflammation in CD [94,95], while some other studies showed that MR enteroclysis offers much better detection of mucosal abnormalities then MR entergraphy [96]. Hence, the choice should be some tradeoffs based on some factors including detection sensitivity, patient acceptance, the intestinal segmented to be examined, the suspicions, and a cost-benefit analysis. MRI enteric contrast agents are generally classified based on their signal intensity on T1- and T2-weighted images [93,97] These agents can be divided into categories: biphasic contrast agents [98,99,100,101,102,103,104,105] (including water, polyethylene glycol gadolinium chelates, locust bean gum, methylcellulose, mannitol, barium sulfate, manganese) that exhibit low signal intensity on T1 and high signal intensity on T2 images; negative contrast agents [106,107,108,109,110] (including ferumoxsil oral suspension, oral superparamagnetic particles, and perluxorooctyl bromide) that show low signal intensity on both T1 and T2 images; and positive contrast agents (including gadolinium chelates, manganese, blueberry juice, pineapple juice, and food substances) [3,109,111] that demonstrate high signal intensity on both T1 and T2 images. Recently, inorganic nanomaterials with rapid clearance have emerged as a promising approach for developing enteric contrast agents for MRI [112].

#### 2.1.5. Others: Ultrasonography and Scintigraphy Image for IBD

In addition, ultrasound studies were also used to investigate IBD in a non-invasive, relatively low-cost, easily accessible manner [113,114], especially for imaging IBD in pediatric patients [115], providing rapid evaluation information on transmural changes, intestinal wall stratification, and the sites of involvement in IBD [116,117,118,119]. Ultrasonography was also shown to accurately detect bowel wall thickening involved in IBD [120,121] and its complications [122] and to assess the actual activity [123]. However, ultrasound is not commonly used in the USA, whereas in Europe it is often used to evaluate the conditions of the small bowel. A study was performed to test the accuracy of transabdominal bowel sonography (TABS) against surgical findings; 213 patients with Crohn’s disease were included in the study, and 33 of them underwent their respective bowel surgery and were further evaluated. Out of these 33, TABS displayed at least one complication in 32 of the patients. The remaining 180 that did not undergo surgery were not further assessed. In 12 out of 180 of these patients, TABS showed intestinal complications, including strictures and fistulas. TABS did not show any complications in the remaining 168/180 patients. For the 33 that underwent surgery, TABS was able to identify strictures in 22/22 of the patients and exclude them in 10/11 patients, resulting in 100% sensitivity and 91% specificity. Fistulas were correctly identified in 20 out of 23 patients and ruled out in 9 out of 10 patients, resulting in a sensitivity of 87% and a specificity of 90%. Abscesses were correctly identified in all 9 patients who had them and ruled out in 22 out of 24 patients who did not, resulting in a sensitivity of 100% and a specificity of 92%. Overall, TABS proved to be a highly sensitive imaging modality for Crohn’s disease [124]. Accordingly, the 2019 ECCO-ESGAR guideline acknowledges intestinal ultrasonography as a prospective approach for the diagnosis and monitoring of IBD [125]. Furthermore, some researchers also suggest that the application of point-of-care ultrasonography for the IBD patients’ assessment in the emergency department may expedite both diagnosis and treatment, as well as minimize the need for supplementary imaging modalities [126].

Additionally, leukocyte scintigraphy is occasionally reported in preclinical studies to detect the inflammation caused by the white blood cells migrating to the intestinal tissue [62,127]. 99mTc-HMPAO (hexamethyl propylene amine oxime)-labeled leukocyte scintigraphy can aid in both PET/CT imaging when the modalities lack clarity, and to help determine an appropriate treatment or therapy program for previously diagnosed IBD. Technetium can identify the location of the inflammation and assess its activity level. Tc-labeled scintigraphy has proven to be extremely useful due to its various advantages, including having no side effects, having high sensitivity and specificity, low invasiveness, and the ability to perform even during the early phases of the disease and without requiring a patient to empty their bowels [128]. A study involving 85 suspected pediatric patients with inflammatory bowel disease (IBD), with a mean age of 12.4 ± 4.3 years and 47% boys, underwent scintigraphy using 99mTc-HMPAO-labeled leukocytes. The results were compared with the final diagnosis determined by endoscopy, histology, and other imaging methods. Scintigraphy results were consistent with the final diagnosis in 78 (91%) patients, resulting in a sensitivity of 89%, a specificity of 91%, and an accuracy of 91%. The interobserver agreement was 0.82, with a radiation dose estimate of 4.2 ± 1.5 mSv [129].

### 2.2. Appendicitis

The appendix, often referred to as a vermiform appendix, is a blind-ended loop located near the beginning of the large intestine, typically with the size of a finger (2–8 inches) [130]. The appendix provides no known function for the human body but is believed to relate to the immune system and digestion of cellulose in a historic period before modern human time [131]. Appendicitis occurs when the appendix becomes obstructed (by fecalith, fecal matter or lymphoid hyperplasia) and inflamed, followed by the build-up of mucous around the site, resulting in an increased amount of pressure. In the USA alone, each year there are at least 300,000 new cases of appendicitis [132]. Clinical features of appendicitis include diffuse abdominal pain, nausea, anorexia, and vomiting. Sometimes, it can lead to bacterial infection that can cause the appendix to swell and pain in the right lower quadrant [133]. Appendicitis is a frequent cause of abdominal pain that often necessitates surgical intervention in both adult and pediatric populations. Early diagnosis is crucial for appendicitis. If appendicitis goes without diagnosis and proper treatment, rupture, abscess formation, peritonitis, and even death can occur [134].

#### 2.2.1. CT for Appendicitis

**Figure 4:**
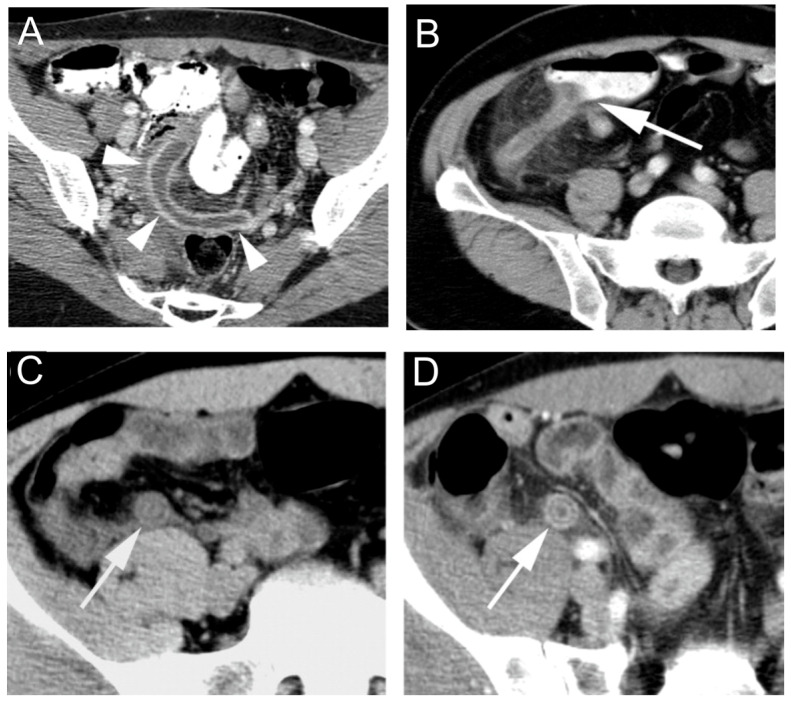
Computed tomography findings of appendicitis. (**A**) Axial CT image showing an enlarged, fluid-filled appendix (arrowheads). (**B**) Axial CT image showing edema of the cecal tip with oral contrast pointing (arrow) towards the base of the inflamed appendix. Axial CT images in the same patient (**C**) before and (**D**) after intravenous contrast show a thickened appendix with submucosal edema. Reprinted with permission of [138].

The most common imaging techniques include ultrasound and CT as well as others including MRI, barium enema study, and nuclear medicine imaging [20,135]. Computed tomography is a more precise and reproducible imaging method for appendicitis [136,137]. Enlargement of the appendix is a hallmark of acute appendicitis and others may include wall thickening, mural hyper-enhancement (Figure 4A), inflammation of the cecum (Figure 4B), and stratification (bull’s eye or target sign) (Figure 4C,D) [138].

In the past several years, there have been several advancements in approaches to CT imaging of appendicitis, including (1) helical CT using intravenous or oral agents; (2) focused appendicular CT imaging method for the right low quadrant only; and (3) unenhanced CT of the abdomen and pelvis [139]. A study evaluating CT scans of 89 patients with suspected acute appendicitis found that radiologists missed 93% of perforations that were later diagnosed by pathology. The radiologic diagnosis of perforation was only reported in 9% of cases, with a sensitivity of 37.5% and a specificity of 62.5% for accurately identifying perforation in non-perforated cases. No secondary finding had clinically reliable sensitivity or specificity to predict perforated appendicitis [140]. This study suggests that while CT may be useful for determining perforation in acute appendicitis, further methods to improve precision should be explored to guide treatment options. Oral contrast media including barium sulfate solution or iodinated solution will be used 1 to 2 h prior to the helical CT scan. If the patients have difficulty drinking the contrast medium, administration can be done by a nasogastric tube [131]. The most common contrast method enables us to cover and enhance the entire abdomen and pelvis [141]. This well-established technology can provide high sensitivity (96%), enhanced specificity (89%), and good accuracy (94%), whereas the delay is the main disadvantage of the method as patients need to wait for at least 1 h after oral contrast administration [142]. A more focused method put forward by Rao et al. used rectal contrast medium and scanned only the lower abdomen and upper pelvis [143]. It was shown that the sensitivity, specificity, and accuracy proved to be as high as 98%. Additionally, compared to helical CT with the administration of contrast materials by mouth and through the colon, this more focused method only through the colon could be performed immediately and bypassed the potential risk or discomforts from oral or IV administration. Further, Wise et al. made a comparison between the focused technology and the unfocused one and found that the accuracy of both is very similar, but focused CT stood out with aspects of the interpretation in positive cases for radiologists [144]. On the other hand, however, some other work showed that unfocused CT could establish more cases and allows for a more complete examination, whereas the focused one missed several diagnoses where immediate surgeries were needed. Additionally, unenhanced CT of the abdomen and pelvis protocol was also used as it can avoid the side effects induced by the contrast medium. Although the unenhanced method can provide the desired accuracy as well, the overall usefulness is still questionable, especially in the cases of children and women [145,146,147,148]. Interestingly, LDCT has been shown to be comparable to conventional dose CT in terms of clinical outcomes and diagnostic performance [149,150].

#### 2.2.2. Ultrasound of Appendicitis

Ultrasound is another method for the diagnosis of appendicitis that was first demonstrated by Deutsch and Leopold in 1981, and then further developed by Puylaert in 1986, known as the graded compression method [151]. Typically, an appendix with inflammation is non-compressible and enlarged, while a normal appendix is usually compressible with layered structures [138]. During an ultrasound procedure, the appendix wall is first assessed for inflammation. This is a crucial step, but it is not the only criterion for diagnosis. The appendix must be further assessed in the transverse plane for thickening of the muscular wall and an increase in diameter as well as peristalsis and compressibility. The entire length of the appendix must be assessed because it is often that only certain portions, typically the appendiceal tip, show more pronounced inflammation [131]. The sonographic detection method should only be used as a supplementary imaging method. Usually, an enlarged appendix diagnosed by ultrasound is not conclusive and other findings are required to confirm the diagnosis of appendicitis [152,153]. On the other hand, compared to CT, the most important strength is its absence of ionizing radiation exposure, especially for children and pregnant patients [154]. The other strengths of ultrasound for appendicitis imaging include the low cost, easy patient preparation and real-time evaluations [152]. On the one hand, it was shown that the ultrasound method could offer high sensitivity. Kaneko et al. studied 165 children to evaluate the accuracy of the ultrasound method for decision making in the treatment of acute appendicitis [155]. An appendix with a diameter of more than 6 mm was diagnosed with appendicitis and severity was categorized into four grades based on intramural structure. The authors concluded that not only can ultrasonography image all inflamed appendices but also can predict the severity of disease with high sensitivity. On the other hand, cases of acute appendicitis diagnosed using ultrasound showed a low diagnostic accuracy in other retrospective studies [156,157]. Interestingly, the diagnostic specificity of ultrasound for appendicitis appears to be contradictory, with some studies demonstrating high specificity ranging from 90% to 100% in differentiating perforated from non-perforated appendicitis in children [158], while others reported lower specificity values (33.33%) in a mixed population of adults (25) and children (47) [157]. These studies suggest a moderate correlation between age, appendicitis types, and diagnostic specificity. Another study evaluated the diagnostic accuracy of magnetic resonance imaging (MRI) and ultrasound (US) in cases of non-visualized appendicitis in pediatric patients [159]. A retrospective review summarized the medical records of 589 pediatric patients who underwent MRI and/or US for appendicitis, showing that MRI had a 100% accuracy rate for non-visualized appendicitis without secondary signs, while US had a 91.4% accuracy rate. However, when there were secondary signs of appendicitis, the accuracy rate dropped to 50% for MRI and 38.9% for US. MRI is effective in excluding appendicitis even when the appendix is not directly visualized. Otherwise, negative US without direct visualization of the appendix was less useful [159]. The study also showed a moderate correlation between US and MRI results.

#### 2.2.3. MRI for Appendicitis

MRIs are beneficial for patients who are pregnant, particularly in their first trimester, because of the extremely low risk of radiation. It is especially crucial for pregnant patients with appendicitis to undergo an accurate diagnosis, and MRIs are recommended for use as a first-line diagnostic test [160,161]. MR imaging is known for superior anatomic resolution in conjunction with no ionizing radiation, as noted previously. The appearance of appendicitis with MR imaging consists of an enlarged appendix filled with fluid and inflamed, and/or with abscess formation [138]. A study was carried out to test the accuracy of ultrasonography and MRI compared to CT imaging. The study consisted of patients first tested with CT imaging and another group where patients initially tested with ultrasound and then an MRI. CT and MRI were read as negative if there were no signs of inflammation near the cecum and ileum and the appendix appeared normal. Ultrasonography was read as negative only if the appendix appeared completely normal. The results proved that an ultrasound followed by an MRI proved to be equally as accurate as a CT [162]. Further studies were conducted to assess the rate and risk of non-visualization of the appendix and alternative diagnoses using MRI for suspected appendicitis in pregnant women [163]. The study enrolled 171 pregnant women who underwent MRI for suspected appendicitis, revealing that the rate of non-visualization of the appendix on MRI was 30.9%, with a higher likelihood of non-visualization in women beyond the first trimester. Among the remaining 118 women with a visualized appendix, 18 had imaging findings consistent with appendicitis, and 12 cases of appendicitis were confirmed on pathologic evaluation. Additionally, MRI provided diagnoses for other diseases in 74 women (43.3%) and guided further management with alternative diagnoses. Among the 43 women who had a nondiagnostic ultrasound prior to MRI, the subsequent diagnostic MRI rate was 65% (*n* = 28) [163]. The study found that MRI has a high diagnostic rate and accuracy in pregnant women with suspected appendicitis, and it can also provide alternative diagnoses.

#### 2.2.4. Other

Abdominal radiographs can indicate obstruction, perforation, and infection, but it is not that reliable. Barium enema examinations are also occasionally used but are not very reliable, because in 15% of patients, the appendix does not fill with barium [131,135]. Nuclear medicine imaging is an alternative method that was approved by the FDA in 2004 for scintigraphic imaging. The radiopharmaceutical contrast agent technetium Tc 99m-labeled fanolesomab kit binds to neutrophils found at infection sites; 90% sensitivity and 87% specificity for detecting acute appendicitis have been achieved by using this contrast agent. Nuclear medicine imaging is also excellent in ruling out appendicitis and preventing unnecessary surgery, in addition to confirming that surgery was required, as well as distinguishing the difference between Crohn’s disease and appendicitis [131].

### 2.3. Carcinoid Tumors and Colorectal Cancer

Neuroendocrine tumors consist of heterogeneous groups of abnormal tissues originating from cells in the endocrine system. The endocrine cells are usually formed from the adrenal medulla, the pituitary, parathyroids, and islets in the thyroid or pancreas [164]. These tumors most commonly occur in the gastrointestinal tract, particularly in the small intestine and appendix, but they can also occur in the lungs and other organs. They rarely occur in the general population and typically progress slowly, often over years, leaving patients asymptomatic until the tumors have grown to a significant size [165]. When symptoms do occur, they may include abdominal pain, diarrhea, flushing, and wheezing. One of the main characteristics of carcinoid tumors is the synthesis and secretion of serotonin, which is metabolized to acid and excreted in urine [166]. It can be difficult to confirm a malignancy, and often cannot be done without the detection of a lymph node invasion or distant metastases [167]. On the other hand, colorectal cancer (CRC), specifically, affects the colon and rectum, which are integral parts of the digestive system. CRC is the third most commonly diagnosed cancer in both men and women worldwide, with approximately 1.9 million new cases and 935 thousand deaths estimated in 2020 [168]. The incidence of CRC has been increasing in many countries, particularly in Asia and Africa, due to changes in diet, lifestyle, and aging populations. CRC is a heterogeneous disease that arises from the accumulation of genetic and epigenetic alterations, leading to the transformation of the normal colonic epithelium into adenomas and eventually invasive carcinomas [169]. Early detection of CRC is critical for improving patient outcomes, as it can lead to earlier intervention and better survival rates.

Imaging plays a critical role in the detection and management of gastrointestinal tumors and their metastases including SPECT, PET, CT, and MRI. For in vivo imaging in primary diagnosis and staging of carcinoid tumors, tomographic imaging with radiolabeled analogs of somatostatin (111 In-pentetreotide) has been accepted as standard; however, PET, CT, and MRI have their ways to improve the process [170]. One study on MRI consisted of 29 patients, 12 of whom had MR examinations prior to the biopsy and 17 who underwent imaging postsurgically. Of the 12 who underwent imaging prior to the biopsy, 8 of the patients had detectable primary tumors. The primary tumors had two common and distinguishable morphologies: there was either a nodular mass originating from the bowel wall, or regional uniform bowel wall thickening. As for the patients who had undetected primary tumors, 1 patient had a primary tumor not in the imaging range, while the site of the primary tumor was never established in another patient. As for the other two patients, the primary tumor was initially missed, but then later detected in the terminal ileum and the stomach. From this study, it was determined the majority of malignant carcinoid tumors were located in the lower ileum and right side of the colon [171].

In the TNM staging system for colorectal cancer, the involvement of regional lymph nodes is used to determine the N stage, while the involvement of other nodes is classified as metastasis [172]. The mesorectal nodes are often the first and most frequently involved group, with nodal metastases commonly found within the proximal 5 cm of the tumor [173]. Extramesorectal nodes are typically involved in locally advanced cancers, while inguinal nodal metastases are rare in rectal cancer and associated with a poor prognosis [174]. It should be noted that malignant disease can still be present in very small nodes, and a size cutoff of 5–8 mm is commonly used for diagnosing malignancy. The location of suspicious nodes relative to the tumor and mesorectal fascia should also be evaluated. Magnetic resonance imaging (MRI) with lymph node-specific contrast agents, such as ultrasmall superparamagnetic iron oxide (USPIO), has shown promising results in characterizing lymph nodes [175]. USPIO is a type of nanoparticle that reduces the signal intensity of normal cells on T2- and T2*-weighted imaging. This results in malignant nodes, which do not take up USPIO particles, appearing brighter compared to benign nodes, as well as enhancing relative to normal tissues. In a study of 25 patients with rectal adenocarcinoma, two radiologists independently evaluated mesorectal nodes using morphologic criteria and USPIO enhancement on T2-weighted and T2*-weighted MRI. The sensitivity and specificity of both criteria in identifying malignancy in 126 pathologically matched mesorectal nodes were compared. The use of morphologic criteria had an average sensitivity of 65%, specificity of 75%, positive predictive value of 19%, and negative predictive value of 96%. The use of USPIO criteria resulted in an average sensitivity of 65%, specificity of 93%, positive predictive value of 43%, and negative predictive value of 97%. The use of USPIO MRI resulted in enhanced diagnostic specificity for both observers, with good interobserver agreement observed for USPIO criteria and fair agreement for morphologic criteria. Currently, the only FDA-approved and commercially available USPIO is ferumoxytol [176].

The findings of CT, US, and MRI in advanced carcinoid tumors were frequently compared in the study. It was observed that intestinal tumors tend to metastasize with increased size and invasion. CT typically showed an ill-defined mass of soft-tissue density arising from a thickened bowel wall, while MRI often revealed characteristic features of carcinoid tumors, such as specific shape, ileal location, adhesion, spread to adjacent loops, metastatic liver lesions, and other associated conditions. However, MRI had lower accuracy compared to CT. Ultrasonography was able to detect most major features, including wall thickening, solid masses, and liver metastases, but additional imaging was usually required for confirmation. It was determined upon the conclusion of this study that CT was the most accurate modality [177]. Another systematic review and analysis of published studies was conducted to evaluate the sensitivity of computed tomographic (CT) colonography and optical colonoscopy (OC) in detecting colorectal cancer [178]. The analysis included 49 studies with a total of 11,151 patients and a cumulative colorectal cancer prevalence of 3.6%. CT colonography was found to have a sensitivity of 96.1% in detecting colorectal cancer, while OC had a sensitivity of 94.7%. The study concluded that CT colonography, especially when using both cathartic and tagging agents in bowel preparation, was highly sensitive for detecting colorectal cancer and may be more suitable than OC for initial investigation of suspected cases. In addition, PET imaging was also utilized to visualize carcinoid tumors. A study aimed to evaluate the effectiveness of 18F-labeled anti-carcinoembryonic antigen (CEA) T84.66 diabody, a genetically engineered non-covalent dimer of scFv, for microPET imaging of colon cancer xenografts [179]. The radiolabeling process and biodistribution of [18F] FB-T84.66 diabody were evaluated in athymic nude mice with subcutaneous human colon carcinoma LS174T tumors. The results revealed that the diabody exhibited rapid and high tumor uptake with fast clearance from the circulation in the mouse xenograft model, leading to high-contrast images. These findings suggest that [18F] FB-T84.66 diabody has the potential to be a PET tracer for CEA-producing malignancies, owing to its tumor-specific probes and rapid blood clearance.

**Figure 5:**
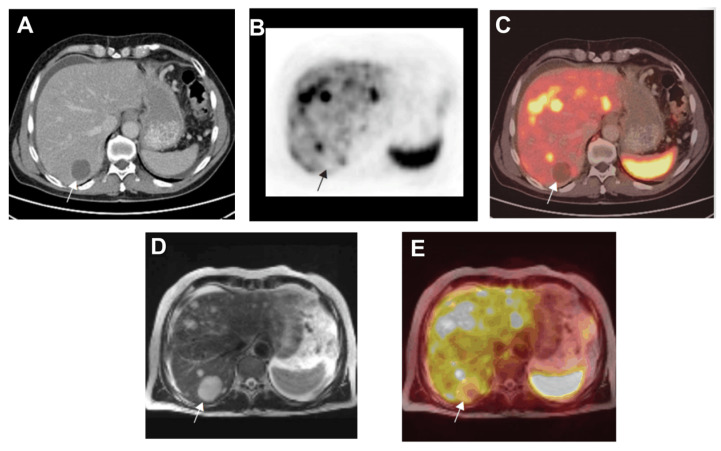
A patient with extensive metastatic disease of an intestinal carcinoid tumor. (**A**) Automatic fused image of PET/CT. (**B**) A venous-dominant contrastenhanced CT scan. (**C**) T2-weighted TSE image of MRI. (**D**) A manual fused image of PET/MRI. (**E**) with multiple hepatic metastases. PET, CT and the overall view of all MRI sequences identified a great cyst in the right lobe of the liver (arrow). Reprinted with the permission of [170].

Another study was carried out to test the efficacy of three radiotracers: 123I-MIBG, 111In-[D-Phe1]-DTPA-octreotide, and 18F-FDG. Out of 15 patients, there were 11 with carcinoids and 4 with paraganglioma, and out of 12 of the 15 patients, no more than 2 out of the 3 imaging modalities indicated positive for abnormal levels of tracer uptake. The intensity of tracer uptake at tumor sites was high at 93% for 18F-FDG, 85% for 111In-[D-Phe1]-DTPA-octreotide, and 62% for 123I-MIBG. In patients regarded as positive, the identified numbers of lesions for 123I-MIBG, 111In-[D-Phe1]-DTPA-octreotide, and 18F-FDG were 67, 44, and 107, respectively [167]. Another study comparing PET, CT, and MRI determined that PET was the best identifier for osseous metastases [170]. Out of all the patients, there were 12 with osseous metastases, and PET alone, PET/CT, and PET/MRI all correctly identified all 12 lesions, while CT only identified 1 and MRI identified 8. Furthermore, PET or PET in conjunction with CT or MRI were all good identifiers of lymph node metastases, with accuracies of 91.9%, 100 %, and 97.3%, respectively. CT alone had an accuracy of 83.8% and MRI had 64.9%; the images are shown in Figure 5. PET, CT, and MRI sequenced identified cysts in the right liver lob as indicated by arrow. Moreover, because of peritoneal extension of the disease, a perihepatic and perisplenic fluid collection was observed by CT and MRI. Next, 111In-pentetreotide single photon emission computed tomography (SPECT) tested against planar scintigraphy was also used to localize abdominal carcinoid tumors. In one study SPECT localized 16 additional tumor sites compared to scintigraphy. Only 21 out of the total 30 liver metastases were seen by scintigraphy. It was concluded that In-pentetreotide SPECT is more suited to the localization of abdominal carcinoids and their associated metastases. It is more sensitive in detecting tumors, in addition to determining liver and abdominal involvement [180].

**Figure 6:**
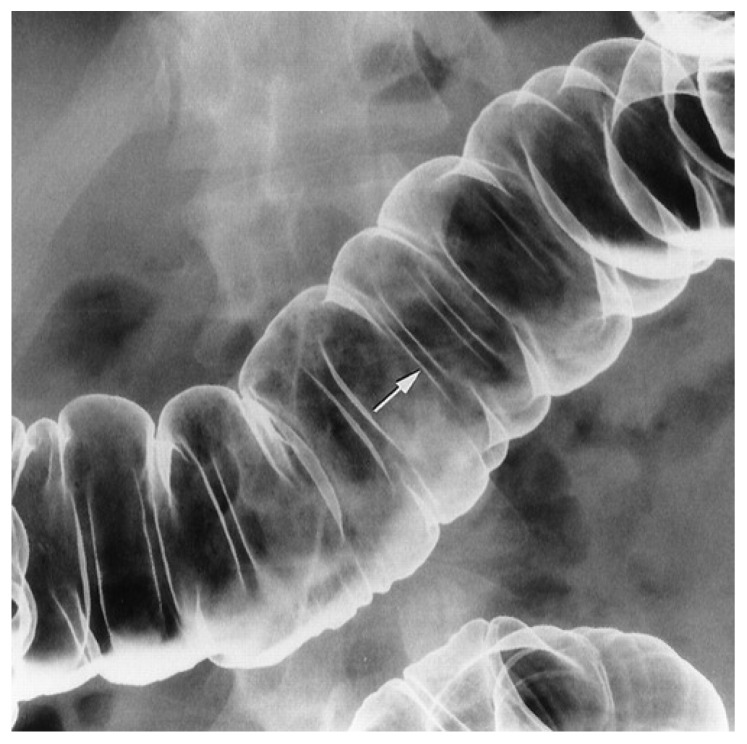
Spot radiograph of the middle of the transverse colon obtained from a patient near-erect position. The interhaustral folds are straight; a representative fold is identified with an arrow. The haustral sacculations are distended, but not overdistended and flattened. Reprinted with permission of [135].

Double-contrast barium enema examination for detecting abnormalities in the colon was reported as Figure 6 shows. It is a flexible examination in which the fluoroscopist interacts with the patient, the controls of the fluoroscope, and the image on the television monitor. The examination creates images of the colon by manipulating the patient, the barium pool, and the amount of air insufflated into the rectum. Fluoroscopy is crucial for guiding the radiologist to obtain spot images with adequate technical factors. The fluoroscopist analyzes the luminal contour, the barium-coated mucosal surface, and the barium pool to detect abnormalities in the colon. It aims to describe and illustrate general concepts in the performance of a high-quality double-contrast barium enema examination. The recent focus on colonic cancer screening has renewed interest in the double-contrast barium enema examination, making it a valuable resource for radiologists and healthcare professionals interested in colonic imaging.

### 2.4. Gastric Diseases

**Figure 7:**
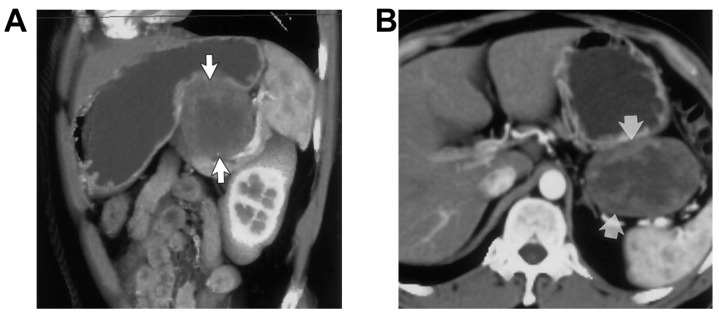
Sagittal (**A**) and axial (**B**) oblique contrast-enhanced 3D volume-rendered CT scans revealed a round exophytic mass in the stomach, 5-cm exophytic mass (arrows) that arises from the stomach, which proved to be a benign GIST during surgery. Reprinted with the permission of [183].

There are many types of gastric diseases including adenocarcinomas, lymphoma, gastrointestinal stromal tumors, gastritis, peptic ulcer disease, emphysematous gastritis, and gastric varices [181]. For simplicity, we mainly discuss the commonly used CT imaging modality, although other imaging/diagnosis methods such as endoscopy and biopsy are primarily used for clinical practice. Adenocarcinoma is responsible for 95% of malignant primary tumors of the stomach [181], remaining the most common gastric malignancy with a 5-year survival rate being less than 20% [182]. Accurate staging of this cancer is crucial for guiding management and involves assessing, for local invasion, as well as nodal and distant metastases. CT is standard for staging because it is most capable of detecting all of these complications (Figure 7) [183,184]. Furthermore, water is often used as an oral contrast agent due to the good gastric distension and visualization of the gastric wall it provides, in addition to its good tolerance. In one study by Hori et al. where water was used as a contrast agent, CT detected 95% of advanced carcinomas and 93% of elevated early carcinomas [185]. When comparing conventional axial two-dimensional (2D) CT to multiplane and 3D spiral CT images, 3D images were able to provide more information and aid in the detection of early and advanced tumors, with a tumor detection rate of 93.5% for 3D imaging and a rate of 64.5% for axial imaging alone [186]. Moreover, the use of 3D multi-detector row CT improved the T and N staging accuracy of gastric cancer, with a T staging accuracy of 84% for 3D imaging compared to 77% for 2D CT images, and an N staging accuracy of 63% for 3D imaging compared to 61% for 2D images, respectively [187]. In particular, the detection rate of early gastric cancer was significantly improved by up to 96% with the use of 3D imaging. Other studies have demonstrated that the combination of tumor markers, demographic data, CT morphological characteristics, and CT value-related parameters can identify gastric adenocarcinoma subtypes with satisfactory diagnostic efficiency [188].

Lymphoma typically presents as diffuse wall thickening on CT, involving multiple regions of the stomach, in contrast to gastric adenocarcinoma [183]. One study determined the average gastric wall thickness for a patient with lymphoma was 4.0 cm [189]. The stomach should be maximally distended, and an oral contrast agent (typically water) and excess air might be used to maximize findings, which might consist of complications including perforation, extragastric extension or fistulization [183]. More research is now focused on imaging lymphomas using FDG-PET/CT multimodality techniques [190,191,192,193]. Sharma et al. evaluated the role of 18F-fluorodeoxyglucose (F-18-FDG) PET/CT in detecting relapse in 39 patients with primary gastric lymphoma post treatment. Results showed the per-patient-based PET-CT had a sensitivity of 96%, specificity of 91%, and accuracy of 93%. The mean lesion standardized uptake value was 5.9 ± 3.1 (2.3–13.6) [194]. Based on these data, PET/CT appears to be highly accurate in detecting recurrence after treatment in patients with primary gastric lymphoma.

Gastrointestinal stromal tumors (GISTs) are uncommon neoplasms that develop from mesenchymal cells in the wall of the gastrointestinal tract and encompass a variety of different classifications including myogenic tumors, neurogenic tumors or less differentiated tumors [195]. GISTs, at CT, vary in size and appearance and cause further complications with growth, including ulcerations as a result of stretching onto mucosa, necrosis, and calcification [196], and CT allows to display of precise localization, shape, size, and malignancy, exophytic/endophytic components, regional and distant metastases as well as risk grade [183,197,198,199,200,201,202,203] (Figure 7). So, CT is used as the primary modality for the initial diagnosis of GISTs, surgical planning, and post-operative monitoring [204]. For example, Wang et al. demonstrate that all of the cystic necrosis or hemorrhage areas of the tumors seen by the naked eye were detected by multi-spiral CT, and the scopes of those areas were consistent with gross pathology [205]. In addition, recently a considerable number of radiomics researchers have used CT images to estimate histopathological risk and genetic mutations as well as patient prognosis [206], which demonstrates the superiority of CT in the diagnosis of GIST.

CT is also used for diagnosing gastritis, particularly in patients presenting with general symptoms such as abdominal pain and nausea. Gastritis typically manifests as thickening of the gastric folds and wall or low attenuation suggestive of inflammation and submucosal edema on CT [181]. However, gastritis can manifest as either diffuse involvement of the entire stomach or as focal or segmental thickening. Gastritis caused by H. pylori infection can resemble neoplasms, as thickening along the greater curvature of the stomach often occurs. Further imaging may be necessary to arrive at a definitive diagnosis, as CT appearances of gastritis and tumors can be similar. The role of 3D CT imaging in gastritis requires further investigation [183].

Peptic ulcers are deep lesions located beyond the muscularis mucosa layer of the gastrointestinal tract that experience exposure to hydrochloric acid and pepsin. Peptic ulcers are usually caused by Heliobacter pylori infection [207]. The infection causes inflammation in the mucosa that results in tissue injury [208]. One method of detecting peptic ulcers is by performing an upper GI endoscopy. Stains are often used with endoscopies, including hematoxylin and eosin. The sensitivity and specificity range from 66–100% and 94–100%, respectively [207]. Another imaging modality used for diagnosis is transabdominal sonography. In one instance, a 90-year-old woman was admitted to the emergency room due to severe abdominal pain and had abdominal sonography performed. The findings showed wall thickening in the pyloric antrum and the presence of an excessive amount of intraperitoneal fluid around the liver. As a result, the patient underwent immediate surgery, which confirmed a complete perforation of the wall of the prepyloric antrum. Sonography is not always the preferred modality, specifically for peptic ulcer perforation. CT scans more accurately demonstrate signs of peptic ulcer perforation [209]. According to one source, the CT virtual endoscopy is a useful tool in diagnosing hemorrhages from peptic ulcer disease. Compared to fiberoptic endoscopy, this method reported a sensitivity of 93% and a specificity of 91%. In another study, angiography was compared to CT. The sensitivity and specificity were 90.9% and 99% [210]. Gastric varices are dilated submucosal collateral veins that occur in the presence of portal hypertension [211]. Multi-slice spiral CT can accurately identify the vascular shape, inflow, and outflow channels of gastric varices [212,213].

### 2.5. Meckel’s Diverticulum

A Meckel’s diverticulum (MD) is a blind sac resulting from the failure of obliteration of the vitelline duct that serves as a means of communication between the yolk sac and the intestine. The obliteration should usually occur during the fifth to sixth week of embryonic development and incomplete obliteration leads to various anomalies including the persistence of a fibrous core or an ileoumbilical fistula. MD is seen in approximately 2–4% of the general population with a ratio of up to 4:1 (men:women). MD is generally asymptomatic, sometimes with obstruction, inflammation, diverticulitis or gastrointestinal hemorrhage, whereas in most cases, obstruction accounts for approximately 40% of symptomatic cases, and can be caused when a bowel loop is trapped by a mesodiverticular band. Gastrointestinal bleeding that is painless is more frequently observed in children, whereas adults tend to experience painful inflammation or obstruction [214,215,216].

**Figure 8:**
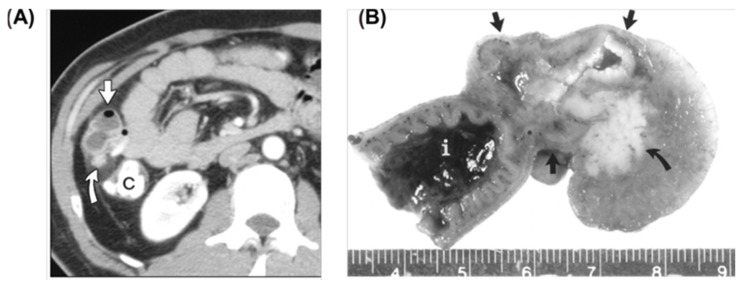
(**A**) Meckel’s diverticulum was diagnosed by contrast-enhanced CT scan indicated by a straight arrow containing air and fluid located in the right paracolic gutter anterior to ascending colon marked by C. Ectopic pancreas was marked by the curved arrow. (**B**) Photograph of the gross pathology specimen demonstrated Meckel’s diverticulum marked by a straight arrow from adjacent ileum marked by i. Solid nodule corresponds to heterotopic pancreatic tissue (shown by curved arrow) that produces adjacent fat. The increments on the ruler are in centimeters. Reprinted with the permission of [221].

The diagnosis of MD has been a challenging task, as various ways are available but with their pros and cons. Plain abdominal radiographs could provide nonspecific information on intestinal obstruction [217]. Unlike appendicitis, inflammation in MD seldom generates gas and fluid levels in the cecum. Conventional small bowel series is not very reliable due to the inability to significantly distend the bowel loops and the visualization of the mucosal fold pattern. Ultrasonography, CT, and magnetic resonance imaging (MRI) may detect complications of MD such as diverticulitis or obstruction, but their overall screening sensitivity is low [218]. Although its role is limited, ultrasonography is commonly used to evaluate children with right abdominal pain. High-resolution ultrasonography can reveal Meckel’s diverticulum as a fluid-filled structure in the right lower abdomen, exhibiting typical gut characteristics such as a blind-ending and thick-walled bowel loop connected directly with the normal small bowel loop [219]. CT has played a limited role in the assessment of MD due to the difficulty of differentiation of MD and adjacent small bowel, but there are still some investigations of CT finding for MD diagnosis [215,220,221,222]. Meckel’s diverticulum exhibits an irregular and thickened vascular wall accompanied by ectatic mesentery, which is surrounded by inflammatory changes in the mesenteric fat as visualized on computed tomography (CT). Furthermore, peripheral abscesses, hydrops, and swollen lymph nodes could be observed [223]. One representative CT scan was shown in Figure 8. Reflux of barium for a colon enema can be occasionally used but is not considered a primary imaging method [215]. Radiographic enteroclysis is one of the reliable modalities. With enteroclysis, it is much easier to obtain consistent distension of bowel segments, and therefore it is easier to detect an abnormality. In total, 415 enteroclyses were carried out over the course of 2.5 years in one study [224]. During the beginning of any procedure, a suspension of barium contrast is infused via a tube in the proximal jejunum or distal duodenum [225]. A Meckel diverticulum has been seen as early as 10 min after the beginning of the infusion. In one specific study, there were 13 surgically confirmed cases of MD, and 11 of the 13 were properly diagnosed with an abnormality detected by enteroclysis [224]. Another preoperative method includes isotope scanning, which is widely used in the detection of bleeding [219]. If the MD contains ectopic gastric mucosa, it will be detected. First, technetium pertechnate is injected intravenously. Gastric mucosal cells take up the contrast and excrete it so that it is visible. Although sensitivity and specificity is only about 63% for adults using this method, it is 90% accurate for pediatric patients. However, false positive diagnoses often occur because of other conditions including but not limited to small bowel obstruction and acute appendicitis, so patients often undergo surgery for a definitive diagnosis [214].

The nuclear medicine method is another notable method of diagnosing MD. 99mTc-pertechnetate is taken up by the mucus-secreting cells of tissues in the diverticulum [226,227]. Using scintigraphy on children resulted in an 85% sensitivity, 95% specificity, and 90% accuracy. For adults, however, the sensitivity was 63% and the accuracy was 46%. This was because gastric mucosa is less often present in patients older than 30. The reported incidence of ectopic mucosa varies from 15–50%, which contributes to the reduced sensitivity. Nevertheless, the sensitivity of scintigraphy can be improved by using H2 antagonists, pentagastrin, or glucagon [228]. A small bowel follow-through with fluoroscopy showed a diverticulum with ulceration [229]. Additionally, another study using PET/CT method was also reported [230]. For pediatric patients, scintigraphy with 99mTc-pertechnetate proves to be the most useful imaging technique for diagnosis. For adults, there is no distinct modality significantly superior to others, but depending on the symptoms, radiographs, enteroclysis and fluoroscopy with barium, and nuclear imaging can all be suitable modalities.

Endoscopies, including small-bowel capsule endoscopy (SBCE), device-assisted enteroscopy (DAE), or double-balloon enteroscopy (DBE), are diagnostic methods for Meckel’s diverticulum. SBCE has become a standard examination for suspected small-bowel hemorrhage after negative bidirectional endoscopies [218]. It has been shown that DBE is an effective tool to diagnose MD with a positive rate of 68.1 [231]. According to clinical features and accessibility, imaging such as MRI and CT is complementary to endoscopy in diagnosing MD [232]. DAE, despite being invasive, is often used as a second-line diagnostic tool in patients with suspected small-bowel hemorrhage, and has shown high sensitivity and specificity for MD [218].

### 2.6. Intestinal Obstruction

Bowel obstruction, which refers to a blockage in the small or large intestine, can disrupt the normal flow of food or other contents through the gastrointestinal tract. Untreated bowel obstruction could result in renal failure or shock [233]. Common causes of blockage include functional ileus and mechanical obstruction [234]. Functional ileus occurs when the bowel loops cannot propagate peristaltic waves. Some forms of functional ileus only affect one or two loops of the bowel, which are classified as localized ileus, while others affect all of the small and large loops of the small bowel. This type of functional ileus is called generalized adynamic ileus and is more common and does not require surgical procedures [234]. However, mechanical obstruction does require surgical intervention [233]. About 80% of mechanical obstructions are caused by hernias and adhesions post surgery. Malignancies, gallstones, strictures, volvulus, and intussusception can also cause mechanical obstruction of the bowel. Additionally, 10–28% of patients with GI cancer may develop bowel obstruction, and 20–50% of patients with ovarian cancer may exhibit symptoms of bowel obstruction [235].

Imaging is crucial in diagnosing and treating bowel obstruction. The small bowel follows through and enteroclysis are both used for assessing the small bowel; however, there are limitations to both methods. Abdominal radiography is often used to test for small bowel obstruction (SBO) and large bowel obstruction (LBO) [236]. Dilation in multiple loops of the small bowel would indicate SBO and dilation in the colon would indicate LBO. In addition, a lack of air and stool in the distal colon and rectum indicates mechanical obstruction [236]. Another study proposed a diagnostic approach for intestinal obstruction using X-ray images, the Hough transform, and possibilistic C-means (PCM) clustering algorithm. The method involved identifying air-fluid levels in the area of large bowel obstruction (LBO) based on clear horizontal linear patterns observed in X-ray images, while small bowel obstruction (SBO) areas showed unclear air-fluid levels. The approach successfully detected intestinal obstruction and demonstrated superior performance compared to anteroposterior abdominal radiography, which has limitations in diagnostic accuracy due to interpreter variability, limited etiological information, and overlapping abdominal structures [237]. It was proved that this method performed effectively in the detection of intestinal obstruction, although the preferred imaging modality for intestinal obstruction is anteroposterior abdominal radiography. Its role in diagnosis is quite limited due to the differences among interpreters, the limited information it provides about the etiological factor of intestinal obstruction [238], and the overlap of abdominal tissues and organs in abdominal radiography [239]. Magnetic resonance imaging does not currently play a large role in the detection of obstruction, but it remains investigational because of the costs of CT scanning. However, it does detect the location and cause of obstruction, and will have a future role in testing for SBO associated with strangulation because it can demonstrate vessels, determine blood flow, and assess oxygenation [236]. In recent years, more and more findings have shown that fetal MRI has diagnostic potential for fetal intestinal abnormalities [240]. According to the changes in MRI signals in multiple sequences, the level of intestinal obstruction can be accurately determined and the possible cause of intestinal obstruction can also be qualitatively diagnosed [240]. Additionally, MRI could also be used to diagnose pregnant patients with intestinal obstruction considering the radiation risk of radiological workup to fetuses, though maternal small bowel obstruction during pregnancy is extremely rare [241].

Another sensitive imaging for bowel obstruction is computed tomography. It is most effective in high-grade obstruction, but it can detect various levels of obstruction, the location of transition points, and the presence of closed-loop obstruction and complications [238], in addition to the causes of the obstruction. Based on the meticulous interpretation of images, CT findings may be pivotal in determining surgical intervention [238]. In another study, Afzal et al. found the sensitivity, specificity, positive predictive value, negative predictive value, and diagnostic accuracy of multidetector computed tomography for the diagnosis of intestinal obstruction were 98.39%, 65.22%, 93.85%, 88.24%, and 93.20%, respectively [242]. The oral contrast agent could be 1.2% barium sulfate suspension 30–45 min before the CT scan [243]. Intravenous contrast agents or fluid in the bowel already sometimes are used. In order to diagnose SBO, the dilated small bowel loops must be larger in diameter than 2.5 cm from the outer to the inner wall [244]. The flow of contrast is assessed to detect low to high-grade partial SBO, with low grade showing sufficient contrast flow through the obstruction, and high grade showing a delay of the contrast flow. For cases of LBO, a CT scan might detect intraperitoneal air to identify associate lesions and the absence of contrast materials in the rectum would indicate complete obstruction; therefore, it is better not to administer contrast agents in that area.

Ultrasound imaging also provides useful information about bowel obstruction. It is used to differentiate between mechanical and functional intestinal obstruction, the site and the cause of obstruction [233]. Blood flow within the wall of the intestine can be assessed because of the color and power of Doppler. Patients could be examined in a supine position without special preparation. The colon, upper and lower abdomen, pelvic cavity, and central region are all scanned for dilated bowel loops and thickened intestinal walls [233]. The sonographic findings of bowel obstruction include dilated fluid and dilated loop that might have thickened the wall and increased the to-and-fro motion of the contents in the bowel [244,245]. Guo et al. demonstrated the accuracy of the ultrasonic diagnosis of intestinal obstruction was 91%, and the accuracy of the ultrasonic etiological diagnosis of intestinal obstruction was 84% in a retrospective study of neonatal intestinal obstruction between 2009 and 2022 [246].

PET, in conjunction with CT, is also used for testing for small bowel obstruction. Given that intestinal obstruction commonly occurs in patients with malignant cancers, some studies have been conducted to assess whole-body PET/CT in the context of gastric cancer [247]. PET with 18F-fluorodeoxyglucose (FDG) helps detect the usage of glucose by malignant cells and is very effective for diagnosing gastrointestinal tumors. The patients were scanned using PET/CT imaging scanner after fasting for 6 h, followed by the examination of glucose level in blood. FDG is administered intravenously, and whole-body imaging is performed 1 h later, with low-dose CT imaging used for attenuation correction. Additionally, an oral contrast agent is given to all patients for PET/CT imaging. FDG uptake in the bowel indicates a positive finding for small bowel obstruction when combined with bowel wall thickening. PET/CT has been demonstrated to effectively identify the causes of bowel obstruction in patients with gastric cancer and aid in surgical management planning [247].

Indocyanine green (ICG) fluorescence imaging has the potential to become an important tool in laparoscopic surgery for strangulated intestinal obstruction. Its role in evaluating intestinal blood flow (IBF), a critical factor in assessing the need for bowel resection after strangulation, has been studied [248]. In vivo, ICG binds to plasma proteins and then emits near-infrared fluorescence when excited by near-infrared light [248]. Studies have shown that ICG fluorescence imaging can provide a more objective assessment of IBF compared to conventional methods, potentially avoiding unnecessary intestinal resection. Utilizing this method may improve intraoperative decision making, although further investigation is warranted.

**Figure 9:**
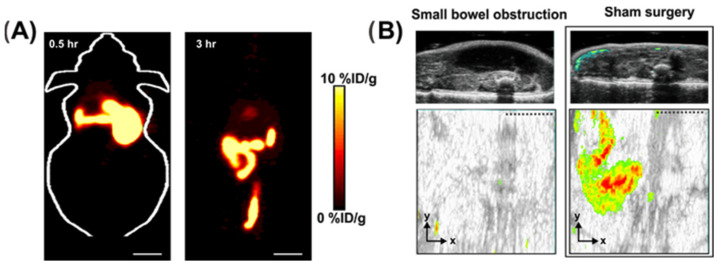
Positron emission tomography (PET) and photoacoustic tomography (PAT) imaging of intestine in mice. (**A**) PET image of intestine using nanonap radio-labeled by ^64^Cu as contrast agent. (**B**) PAT as a diagnostic tool for small bowel obstruction. Reproduced with permission of [14].

Photoacoustic (PA) imaging, a non-ionizing, non-invasive, inexpensive modality has been used for imaging lymph nodes and vessels. Recently, a contrast agent named nanonap, made by pluronic block copolymer and extremely hydrophobic dye napthlocyanine, was developed. This enables the imaging of the intestine as well as the bowel obstruction using the PA modality [14]. Unlike conventional micelles, nanonaps, but not micelles without dyes encapsulated in the core, are kinetically stable, and resistant to dilutions or low temperatures completely. Based on this, nanonaps could be easily purified and concentrated at lower temperatures. In addition, nanonaps can withstand the harsh conditions in the gastrointestinal (GI) tract and safely pass through it without systemic absorption and loss of NIR absorption. Therefore, nanonaps can be used as an ideal contrast agent for photoacoustic imaging of the GI tract with high resolution and low background owing to their high concentration and absorption in NIR and stability in the GI tract [14]. In addition, nanonaps possess Nc intrinsically; thus ^64^Cu could be labeled into nanonaps, which could be further used for positron emission tomography (PET) imaging. This provides another diagnostic tool for small bowel obstruction, as shown in Figure 9.

### 2.7. Pelvic Floor Abnormalities (Constipation)

**Figure 10:**
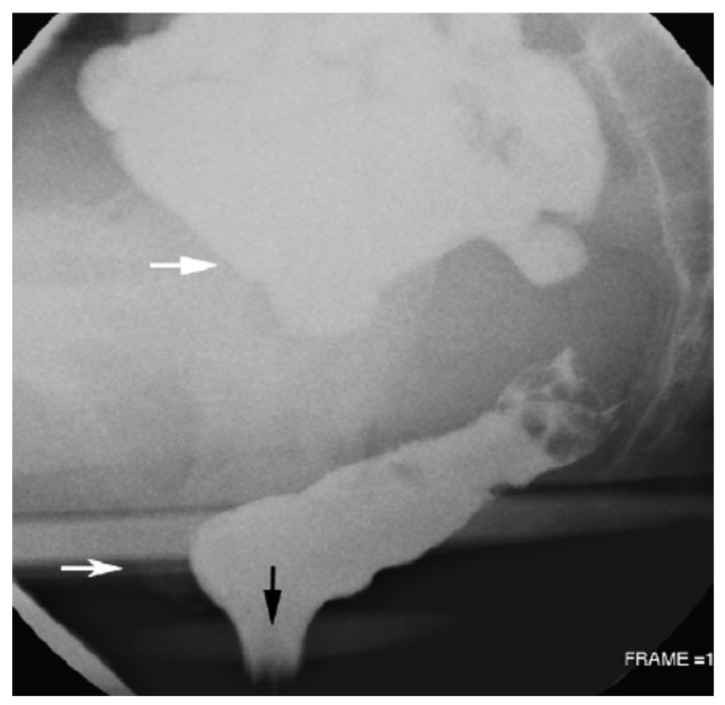
Normal defecography study showing that the anal canal is open marked by a black arrow and has descended below ischial tuberosities (lower white arrow), and contrast-filled small bowel is seen within the pelvis (upper white arrow). Reprinted with the permission of [250].

Constipation is a common gastrointestinal condition, affecting over 15% of the general population living in the Western world [249]. Normal defecation involves a four-step process, including colonic transit, anorectal sensation, expulsion force, and evacuation by the pelvic floor. During rest, the anal canal is closed, and upon squeezing the pelvic muscles, the rectum should rise. Evacuation begins with a descent of the pelvic floor and opening of the anal canal, as shown in Figure 10. When the pelvic floor has any abnormality that prevents evacuation, it is referred to as obstructed defecation [250]. Various imaging modalities are used to assess and diagnose constipation including transit time measurement, conventional and MRI defecography, ultrasonography, and dynamic anal endoscopy [250].

Conventional defecography (also called evacuation proctography) is a medical radiological imaging method for the visualization of a patient’s defecation in real-time using a fluoroscope. It can be used to assess the extent of the rectal obstruction in addition to aiding in finding the cause. This method is usually used in adult and adolescent patients, who can follow the instructions for the dynamic part of the workup [251]. Fluoroscopic images of the pelvic floor are obtained when the muscles are at rest, followed by when they are contracting. The strength of the pelvic floor muscles can be determined in this manner. After the patient has attempted to empty the rectum, either partially, in multiple, 30 s attempts, or completely, if possible, a cine-loop is obtained demonstrating the evacuation [250]. Although the use of digital fluoroscopy dramatically reduces the patient’s exposure to radiation, this imaging technique is not usually tolerated well by patients, and it is unable to detect soft-tissue structures [252]. Another fluoroscopic imaging method used in the diagnosis of constipation is contrast enema, which can provide information about the luminal size of the colon and rectum, the length of involvement, and site(s) of transition in luminal caliber [251]. This method may have both diagnostic and therapeutic effects on constipation, as the contrast agent instilled into the colon and rectum may help reduce fecal impaction [251].

Unlike conventional defecography, MRI defecography can bypass the projectional limitation of fluoroscopic defecography [253]. It is capable of evaluating the pelvic floor in different orthogonal planes and depicting perirectal soft tissue, rectoceles, and intussusceptions more clearly. Patients could be in a sitting or supine position. No administration of contrast media in the vagina or the bladder is needed, but for defecographic studies, contrast agents are needed, including ultrasound gel and mashed potatoes loaded with gadopentetate dimeglumine [254]. MRI defecography can also reveal other pelvic lesions and abnormal content of the peritoneal fornix that may be missed in conventional defecography [255]. Some studies compared conventional radiology and MRI defecography in order to determine the more suitable modality for pelvic floor hernias and other disorders, and it was determined that MRI defecography had a lower sensitivity in, for example, the detection of peritoneal sac herniations [250]. However, it was suggested that less invasive MRI defecography might be a strong candidate in the evaluation of the anatomy of the pelvic floor [256]. The best initial test for the diagnosis of constipation or obstructed defecation is transit time studies, followed by defecography. To complement defecography, MRI or pelvic floor sonography might be used in order to provide additional information about the pelvic floor and its surrounding organs. The cost of MR imaging is greater than that of evacuation proctography; however, it is less invasive and better tolerated by patients, and it is free of ionizing radiation [257].

Ultrasound can be used to evaluate patients with fecal incontinence and the 3D technique can be used to evaluate the pelvic floor. The advantages of this modality include its availability, it is easy to perform, and it does not expose patients to radiation. One disadvantage of ultrasound is its ability to compress pelvic structures such as the bladder, portraying an inaccurate assessment of organs [258]. Radionuclear transit scintigraphy is also useful for categorizing different types of chronic constipation, which allows for different treatments depending on the type. Scintigraphy involves the measurements of the location of an isotope as it goes through the GI tract. For example, when most radioactivity was retained in the proximal colon and transverse colon, the type of constipation is identified as slow transit [259]. The efficacy of abdominal X-rays for diagnosing constipation has been investigated in many systematic reviews and retrospective studies, but using abdominal X-rays to evaluate pediatric patients with functional constipation is not beneficial and may be harmful due to frequent radiation exposure [260]. In addition, studies have shown no diagnostic correlation between clinical symptoms or severity of constipation and abdominal X-ray findings [260].

**Figure 11:**
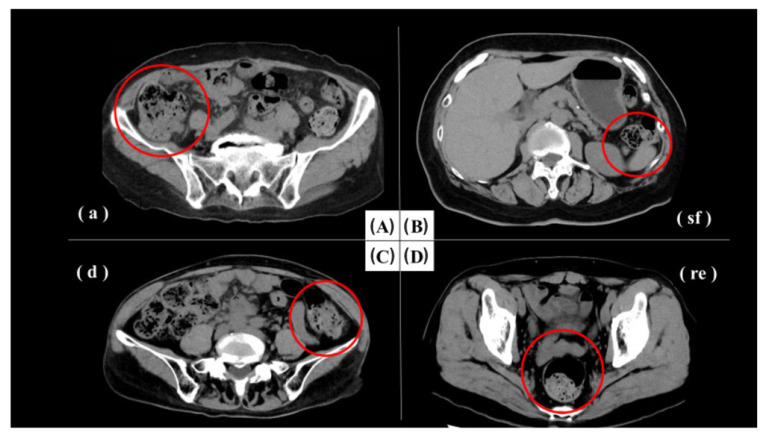
Representative CT imaging for constipation diagnosis. (**A**) CT image was used to rate the stool volume as 4 and the gas volume as 2 (red circle). (a) Ascending colon. (**B**) CT image was used to rate the stool volume as 1 and the gas volume as 2 (red circle). (sf), splenic flexure. (**C**) CT image was used to rate the stool volume as 3 and the gas volume as 2 (red circle). (d) Descending colon. (**D**) CT image was used to rate the stool volume as 3 and the gas volume as 3 (red circle). (re), rectum. Reprinted with the permission of [261].

Computed tomography (CT) is the most important imaging method for accessing patients with known or suspected constipation, especially adult patients. In some cases, CT can also be used in the diagnosis of pediatric patients with constipation via modulating radiation dose [251]. It is readily available, quickly performed, permits the evaluation of latent complications, and allows for structural visualization for extra-colon [251]. CT findings of stool and gas volume, as well as fecal features in different regions of the large intestine, have been correlated with constipation symptoms (Figure 11) [261]. With a sensitivity of 96% and a specificity of 93%, CT has high diagnostic accuracy [251], which is quite high. Therefore, CT is valuable in the diagnosis of constipation and can provide information for clinical management.

## 3. Discussion and Conclusions

This review summarizes the progress in imaging techniques for diagnosing gastrointestinal diseases and overviews the advantages and disadvantages associated with different imaging modalities. Since gastrointestinal diseases include a wide range of conditions that affect individuals across different age groups, histopathological variations have significantly influenced the advancement of gastrointestinal imaging techniques. Specifically, magnetic resonance imaging (MRI) and computed tomography (CT), two of the most critical imaging modalities, have been substantially optimized for gastrointestinal imaging and are still playing a pivotal role in the assessment of gastrointestinal disorders. Extensive research has demonstrated that MR and CT technologies offer advantages in spatial and temporal resolution, as well as improvements in intestinal distention, compared to conventional barium meal fluoroscopy. There has been a significant increase in the utilization of CT scans and MRIs for abdominal imaging in patients with inflammatory bowel disease, with certain demographic factors associated with a higher likelihood of undergoing multiple CT scans. For example, among 176,110 patients with CD and 143,460 patients with UC, there was an increase in patients with ≥1 abdominal CT (CD: +3.6%, UC: +4.9%) per year; similarly, the annual percentage change in patients with ≥1 MRI (CD: +15.6%; UC: +22.8%) also showed an upward trend from 2010 to 2019 [262]. In addition, current research indicates a paradigm shift towards CT for the diagnosis of gastrointestinal cancers.

However, CT examinations involve ionizing radiation, and the long-term use of CT necessitates consideration of the associated radiation exposure dose. Furthermore, CT exhibits suboptimal soft tissue contrast, particularly when visualizing anatomical structures in the nervous system and pelvic region, in contrast to magnetic resonance imaging (MRI). Nonetheless, CT’s principal advantage lies in its rapid scanning speed, which is especially beneficial for clinical emergencies and screening purposes.

FDG-PET imaging reveals the pathological and physiological characteristics of lesions. Ongoing developments include PET techniques based on ^18^F-FSPG PET and Im-munoPET. When the disease is in the early stage, the morphological structure of the lesion area has not yet shown abnormalities, which could not be diagnosed by MRI or CT examination. In contrast, PET can find the location of the lesion and achieve early diagnosis by obtaining three-dimensional images and carrying out quantitative analysis. CT/PET integrates the strengths of CT and PET, such as sensitivity, accuracy, specificity, and precise localization. By providing comprehensive sectional images of the entire body in a single scan, CT/PET enables a holistic understanding of the overall condition, thus facilitating early detection and diagnosis of diseases. However, radiation safety concerns and cost remain barriers to the widespread implementation of PET or CT/PET.

For chronic diseases requiring multiple consecutive examinations to assess disease progression and monitor treatment efficacy, MRI, instead of CT or CT/PET is a preferable option. Compared to CT, MRI offers superior soft tissue contrast resolution, enabling better visualization of inflammatory and fibrotic features within the intestinal wall. MRI has been established as the standard imaging modality for patients with Crohn’s disease, suspected perianal involvement, and perianal fistula conditions. In the imaging of inflammatory disorders in the small intestine, MRI, particularly diffusion-weighted imaging (DWI), is the foremost imaging modality owing to its seamless integration with standard MRI with enhanced disease assessment accuracy. Additionally, advanced MRI techniques have significantly reduced scan duration; notably, the rapid development of advanced MRI pulse sequences enables rapid real-time imaging of the gastrointestinal tract, facilitating the identification of narrow areas and providing valuable information pertaining to peristalsis.

Ultrasound studies provide a swift evaluation of transmural changes, muscle layering, and affected regions. The absence of ionizing radiation is a prominent advantage, particularly for pediatric and pregnant patients, but the accuracy of ultrasound diagnosis is easily influenced by the operator’s experience, examination technique, and care. The 2019 ECCO-ESGAR guidelines acknowledge intestinal ultrasound as a potential tool for the diagnosis and monitoring of inflammatory bowel disease. Photoacoustic tomography (PAT) combines high-contrast optical imaging, spectroscopy-based specificity, and the deep penetration depth offered by ultrasound imaging [263,264]. PAT overcomes the limitations imposed by optical scattering, allowing for satisfactory acoustic spatial resolution at imaging depths of 5-6 cm. Nonetheless, limitations such as the penetration depth impede its translation and need to be solved to render it more suitable for clinical diagnosis and detection [265].

It should be noted that the absence of a common benchmark dataset for gastrointestinal imaging impedes the comprehensive evaluation of different imaging techniques for related diseases. Consequently, the selection of imaging modalities should consider several factors, including detection sensitivity, specificity, accuracy, patient acceptance, the specific segment of the intestine to be examined, suspicion level, and cost-effectiveness analysis.

In summary, this review summarizes the progress of imaging techniques and provides insights into the strengths and weaknesses of various imaging techniques in the context of several representative GI ailments, including inflammatory bowel disease, appendicitis, and Meckel’s diverticulum. The strengths and limitations of each technique should be comprehensively considered when choosing the appropriate imaging modality for the specific clinical scenario. Overall, advancements in GI tract imaging have greatly improved the diagnosis and management of GI disorders. Looking forward, future research efforts should prioritize improving imaging capabilities and targeting efficacy to enhance imaging outcomes. Clinical translation of contrast agents should also be a priority, as many laboratory-developed agents have not yet been applied in clinical settings, possibly due to concerns such as toxicity, complex design, and cost-effectiveness. Exploring strategies involving multiple ligands for multimodal imaging could be a promising approach. Another approach is to develop universal contrast agents that can be used across multiple imaging techniques, thereby streamlining the imaging process and reducing costs. Through concerted endeavors on enhancing imaging capabilities and developing innovative strategies, it is envisioned that more clinical and improved diagnosis choices for GI ailments will be developed for patients and clinicians.

## Data Availability

Not applicable.
